# Nonbacterial Osteitis of the Clavicle: Longitudinal Imaging Series from Initial Diagnosis to Clinical Improvement 

**DOI:** 10.1155/2015/182731

**Published:** 2015-01-27

**Authors:** E. Roos, M. Maas, S. J. M. Breugem, G. R. Schaap, J. A. M. Bramer

**Affiliations:** ^1^Department of Orthopedic Surgery, Academic Medical Center, P.O. Box 22660, 1100DD Amsterdam, Netherlands; ^2^Department of Radiology, Academic Medical Center, Amsterdam, Netherlands

## Abstract

Nonbacterial osteitis is a rare autoinflammatory disease. Often it is mistaken for a tumor or osteomyelitis. We present a case of a twelve-year-old girl referred to our hospital because of a lesion of the right clavicle. The differential diagnoses were sarcoma, osteitis, and Langerhans cell histiocytosis. After biopsy the diagnosis nonbacterial osteitis (NBO) was established. Treatment of choice is a nonsteroidal anti-inflammatory drug. This case report gives a complete follow-up of the disease, showing the pitfalls of the diagnosis.

## 1. Introduction

Nonbacterial osteitis is a rare condition, most often of the femur or tibia, which occurs in children around the age of eleven [1–4]. We present a case in which the onset of the disease coincided with a trauma the entire process and therefore was depicted in imaging and closely monitored, providing a unique overview of the condition.

## 2. Case Report

**Figure 1:**
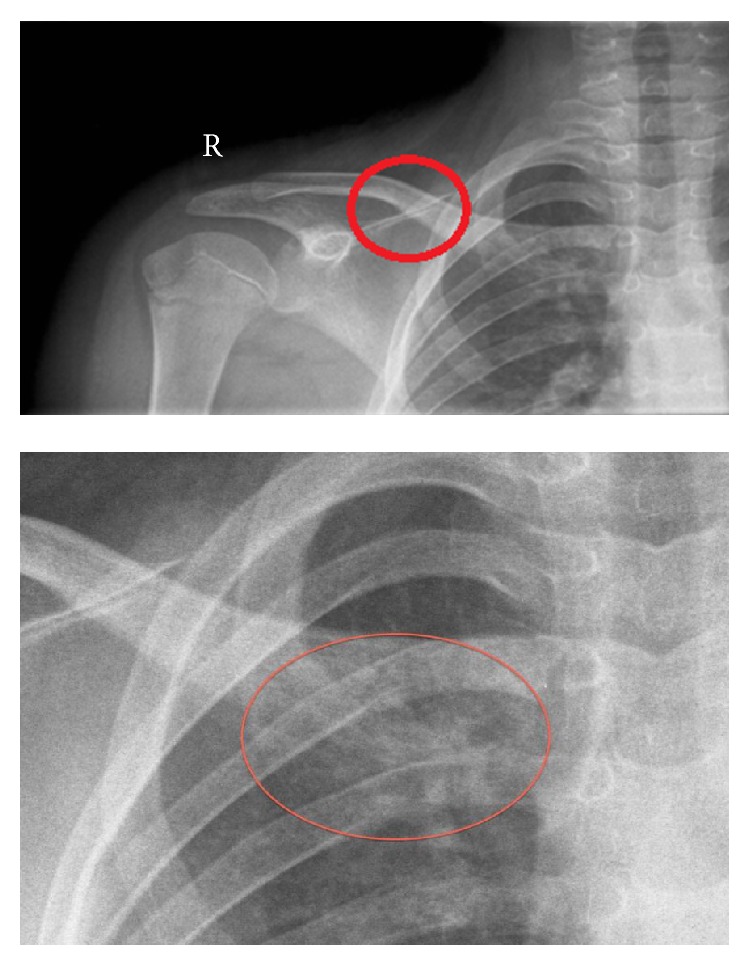
A subtle osteitis is seen. The medial site of the clavicle shows an unclear restriction and a heterogeneous aspect.

A twelve-year-old girl was presented in our bone tumor meeting because of pain and swelling of the right clavicle. Four months earlier in April she had fallen during exercise. She was presented at the emergency room of a hospital in the periphery with a painful right shoulder. An X-ray was made because of a suspected fractured clavicle (Figure 1): in retrospect a minor swelling of the medial clavicle was observed, with a slight heterogeneous aspect. However this went unnoticed at that time. She was sent home with painkillers.

**Figure 2:**
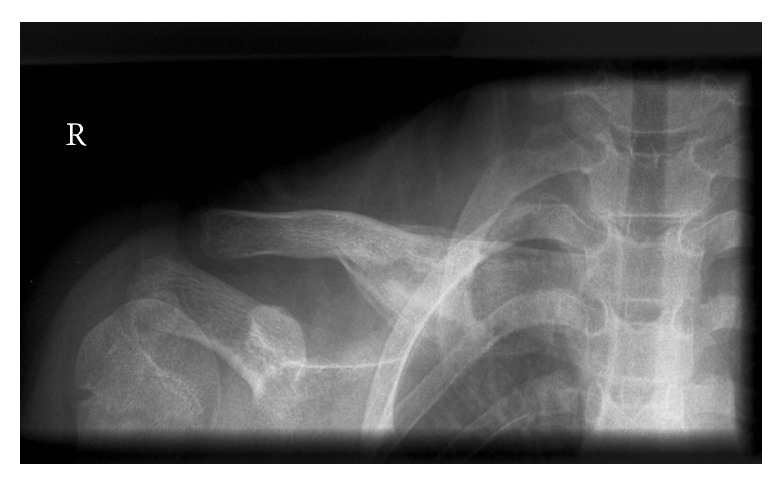
A large mass of the medial clavicle, misread as a callus. It has a moth eaten aspect: typical for osteitis.

Four months later in August, the patient had persistent pain and a swelling of the clavicle during physical examination. A new X-ray was made (Figure 2): this showed a large, heterogeneous mass of the medial side, without a soft tissue component. The bony part had a moth eaten aspect. A lamellated periosteal reaction is observed. This was considered to be callus after the trauma in April. The patient was treated conservatively.

After a few weeks though the swelling increased and pain persisted. Blood tests showed a c-reactive protein (CRP) of 22 and a sedimentation rate (ESR) of 81.

In retrospect especially the moth eaten aspect and periosteal reaction of the lesion were suspect. The differential diagnosis is osteitis/osteomyelitis because of the heterogeneous moth eaten aspect and the localization. The lamellated periosteal reaction however could also be apparent in Ewing sarcoma; however a more lytic lesion and more expansion in the soft tissue usually characterize Ewing sarcoma. Osteosarcoma was also thought of but is very rare in the clavicle and is not distinguished by a lamellated periosteal reaction, but by a more spiculated or sunburst periosteal reaction. The image however could fit Langerhans cell histiocytosis; a rare multisystem disease involving clonal proliferation of Langerhans cells. A moth eaten aspect and lamellated periosteal reaction on X-ray also distinguishes the early phases of the condition.

**Figure 3:**
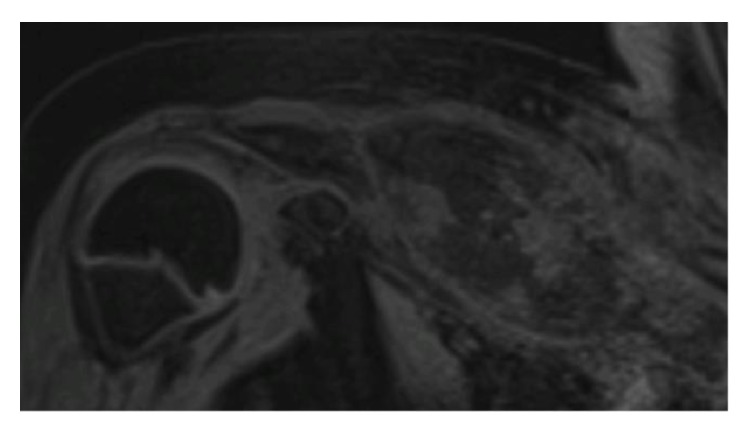
A large bony mass with heterogenous aspect and translucent areas.

It was decided to make a MRI scan to differentiate between a malign or benign origin. On this MRI made in the end of August, we observe a large bony mass, with a heterogeneous aspect and translucent areas. A lamellated periosteal reaction, especially in the superior part, was seen. There seemed to be no soft tissue involvement. The size of the lesion at that moment was 10 × 5 cm. The lack of soft tissue involvement made Ewing sarcoma and osteosarcoma less likely. The absence of osteolysis and a spiculated periosteal reaction attributed to the possibility of a benign origin. An osteitis seemed to be a plausible cause; although Langerhans cell histiocytosis could not be ruled out (Figure 3).

**Figure 4:**
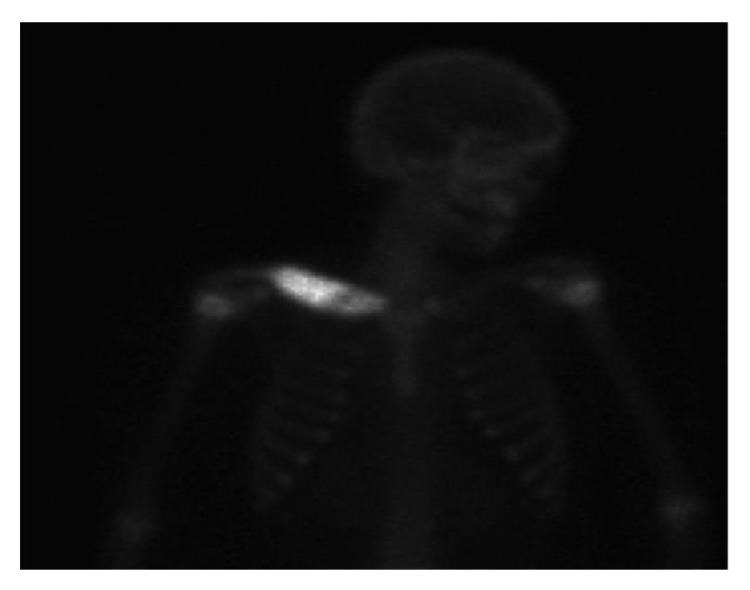
A distinctive hot spot of the right clavicle.

As osteitis is sometimes multifocal a bone-scintigraphy was made on which a hot spot in the right clavicle was seen. Therefor a multifocal disease was unlikely (Figure 4).

The patient was referred to our musculoskeletal referral center, as it was not clear if the lesion was malign or benign.

**Figure 5:**
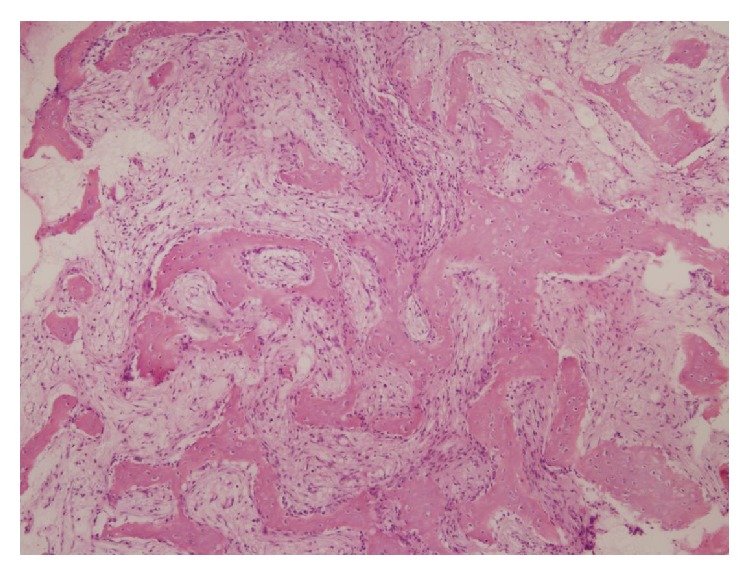


**Figure 6:**
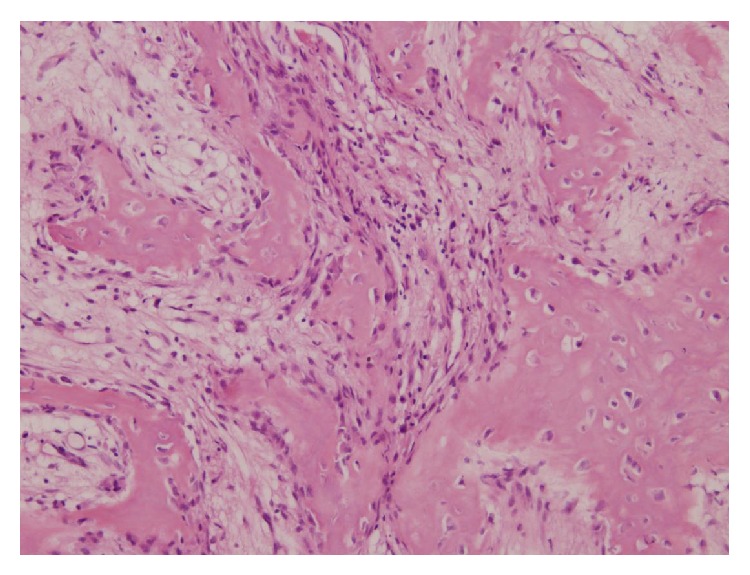


After referral, the radiological material was reassessed. In retrospect the lesion was rather suggestive for osteitis, as is described above in the differential diagnosis. It was decided to do an open biopsy, to exclude malignancy or Langerhans cell histiocytosis. Cultures were taken and tissue was sent for pathological evaluation. Pathological investigation showed existing bone with extensive reactive bone formation along the periosteal surface. In both the trabecular and cortical bone osseous transformation was seen, resulting in a more plexiform aspect of the cortex. The stromal spaces and marrow existed of well-vascularized stroma and especially in the trabecular bone chronic and active inflammation was observed (Figure 5). In the adjacent bone a zone with osteoclast activity and more to the periphery osteoblast activity was seen (Figure 6). No Langerhans cells were observed. Cultures and PCR were negative. Nonbacterial osteitis seemed to be the most likely diagnosis.

Because an unknown bacterial pathogen could not be ruled out completely, antibiotics were prescribed pragmatically, first flucloxacillin for six weeks and then because of relapse a switch to clindamycin for three months. There was not much radiological or clinical improvement. Because of the unsuccessful antibiotic treatment, the negative cultures, and the relatively low infection parameters the diagnosis of nonbacterial osteitis was now confirmed.

**Figure 7:**
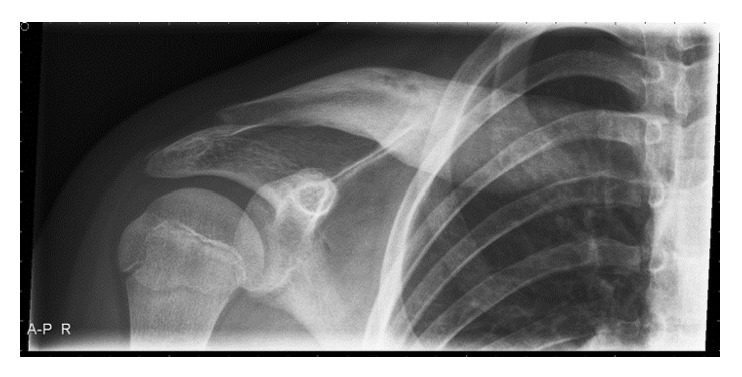
Ossification of the lesion.

According to the reports on the topic nonsteroidal anti-inflammatory drugs (NSAIDs) are the preferred treatment for the condition. Therefor NSAIDs were started [1, 2, 4]. The patient's condition improved dramatically. Inflammation values went down and pain diminished. Eighteen months after the first presentation a follow-up X-ray was made (Figure 7). It showed the same tumor-like mass, which was observed before, but now it had calcified. The moth eaten aspect was gone. This is the natural course of nonbacterial osteitis. At the moment our patient has been free of symptoms for two years.

## 3. Discussion

Nonbacterial osteitis (NBO) is a relatively rare disease [1, 2]. It is a unifocal or multifocal sterile bone lesion, can be acute or recurrent, and is considered to be an autoinflammatory disease [1, 3]. Sometimes it is associated with the SAPHO syndrome (synovitis, acne, pustulosis, hyperostosis, osteitis) and in multifocal and recurrent cases it is called CRMO (chronic recurrent multifocal osteomyelitis) [4, 5]. NBO is considered a diagnosis per exclusionem, because an infectious agent should be ruled out by negative cultures and polymerase chain reaction (PCR) [4, 6].

The median age of presentation is eleven years, with local pain, swelling, and malaise as the leading symptoms [1, 7, 8]. The most common bones affected according to Gikas et al. are the clavicle, femur, and tibia, while Jansson et al. consider the femur, tibia, pelvis, and vertebrae as the most common sites of disease [1, 6, 7, 9, 10].

Imaging shows a characteristic poorly defined, moth eaten, destructive lesion, with expansion, sclerosis, and a solid or multi lamellated periosteal reaction [6, 10]. Sometimes a minor to moderate soft tissue reaction is seen [7]. The absence of an extraosseous mass rules out sarcomas, like Ewing sarcoma and osteosaroma, which are always to be considered in the differential diagnosis [7].

Magnetic resonance imaging (MRI) is regarded as a better diagnostic tool than computer tomography (CT), for it excludes marrow infiltration and soft tissue swelling [7]. Blood tests often show mildly elevated inflammatory markers [1, 7]. In an early stage NBO might look like a callus formation, misleading the clinician, especially when a trauma has preceded the patient's symptoms.

Gikas et al. argue, based on their experience, that a biopsy is no longer necessary. Imaging, clinical, and biochemical findings are sufficient to diagnose NBO [7].

However we still deem biopsy an important diagnostic tool, especially valuable if other symptoms, for example, those of SAPHO, are not present [1, 4, 7, 10].

It is important to determine between malignant, infectious, and autoinflammatory etiology and so biopsy attributes to the choice of a proper treatment [9, 11].

Traditionally antibiotics were prescribed in NBO, based on the thought that there might be an infectious agent involved that cannot be cultured. Though, as it is considered an autoinflammatory disease without an infectious cause, not antibiotics but NSAIDS are considered the best therapeutic choice [1, 4, 7, 10].

TNF-alpha blockage has been described to be successful for the treatment of relapsing cases of NBO [7, 12], supporting the hypothesis that it is indeed an auto-inflammatory disease. Surgery is advised as a last resort, when patients experience significant morbidity despite adequate medical therapy [7].

Because of a minor trauma, our patient was seen at the first aid department and the early signs of NBO were not detected. In retrospect on the X-ray a subtle osteitis is already exposed. We see how the disease has evolved four months later; now the subtle change has transformed in a clear swelling with bone formation and a moth eaten aspect. To differentiate between a malign or benign origin, a MRI scan is made. As there is now extensive osteolysis or spiculated periosteal reaction a malign origin seems unlikely. The imaging is suspect for osteitis. A bone scintigraphy rules out multifocal osteitis, as there is only one hot spot visible. The biopsy and cultures exclude a malignant or bacterial cause. The further course of treatment confirms the diagnosis of nonbacterial osteitis: antibiotics have no effect; however symptoms subside and the lesion calcifies under treatment with NSAIDs, which is the preferred medication.

As far as we know this is the first case with imaging of nonbacterial osteitis in its earliest stages, showing the pitfalls of this diagnosis as the condition can look like a malignancy or an osteomyelitis. One should be aware though that NBO exists, even without the accompanying symptoms of SAPHO. To exclude a malignant or infectious origin, especially if further symptoms of NBO are not present, biopsy is an important diagnostic tool. NSAIDs are the treatment of choice if NBO seems to be the most likely diagnosis. This case provides a complete follow-up of a patient with nonbacterial osteitis, from a subtle start to active inflammation and to calcification once more.
